# Preoperative management and postoperative complications associated with transoral decompression for the upper cervical spine

**DOI:** 10.1186/s12891-022-05081-7

**Published:** 2022-02-08

**Authors:** Wenqiang Li, Bingjin Wang, Xiaobo Feng, Wenbin Hua, Cao Yang

**Affiliations:** Department of Orthopaedics, Union Hospital, Tongji Medical College, Huazhong University of Science and Technology, Wuhan, 430022 China

**Keywords:** Transoral decompression, Upper cervical spine, Complications, Preoperative management

## Abstract

**Purpose:**

This review aimed to describe the preoperative management and postoperative complications associated with transoral decompression of the upper cervical spine, and to clarify the risk factors, related issues and complication management.

**Methods:**

Studies on transoral decompression for the upper cervical spine were reviewed systematically. The preoperative management and postoperative complications associated with transoral decompression for upper cervical deformities were analyzed.

**Results:**

Evidence suggests that preoperative management in patients undergoing transoral decompression for the upper cervical spine is closely related to the occurrence of postoperative complications. Hence, preoperative surgical planning, preoperative preparation, and oral nursing care should be seriously considered in these patients. Moreover, while being established as an effective and safe method, transoral decompression is associated with several postoperative complications, which could be prevented by elaborate preoperative management, improved surgical skills, and appropriate precautionary measures.

**Conclusions:**

The effectiveness and safety of transoral decompression has been improved by the constant development of operative techniques and advanced auxiliary diagnostic and therapeutic methods, with the understanding of the anatomical structure of the craniocervical joint. Therefore, the incidence rates of postoperative complications have decreased. The application of individualized anterior implants and less-invasive endoscopic endonasal approach has improved the effectiveness of transoral decompression and reduced the associated complications.

## Background

Compression of the upper cervical spine is always accompanied by upper cervical deformities, which are known as deformities or impairment of the atlas and/or axis, and their pathogeneses include congenital and secondary deformities such as tumors, infections, and trauma [1–5]. Upper cervical deformities are diagnosed based on clinical symptoms and signs and imaging examinations, including anteroposterior, lateral, and dynamic radiography, upper cervical computed tomography (CT) with three-dimensional reconstruction, and magnetic resonance imaging (MRI). Treatment approaches for upper cervical deformities could be divided into anterior approaches (transoral and transmandibular), posterior approaches (occipitocervical and atlantoaxial fusions), and the combined approach.

The transoral approach can provide direct decompression of the upper cervical spine and improve myelopathy-induced symptoms. The surgical indication for transoral decompression is symptomatic compression of the upper cervical spinal cord caused by upper cervical deformities, including basilar invagination, congenital or secondary pseudarthrosis, malformation or os odontoideum with irreducible atlantoaxial dislocation, inflammatory or infectious diseases, and tumors, among others [1, 2, 6–13]. Preservation of cervical spine movements, earlier rehabilitation, and shorter hospital stay are the advantages of transoral decompression and fixation [14]. Atlantoaxial reduction and efficient decompression could be achieved using a transoral atlantoaxial reduction plate (TARP) for the treatment of atlantoaxial dislocation [15–19]. Moreover, the TARP system could achieve satisfactory clinical results for revision surgery in patients with posterior decompression [20]. However, post-transoral decompression complications are also concerning. Complication rates greatly vary due to the different pathogeneses, associated surgical methods, and sample size. A previous study reported that postoperative complication rates of the transoral approach for a non-tumorous pathogenesis varied from 6 to 21.4% [1, 11, 17, 21]. The complications of transoral approach to treat craniovertebral junction anomalies include infection (4.8%), spine dislocation (4.2%), respiratory complications (4.2%), epidural compressive hematoma (0.7%) and death (0.7%) [1]. The complications of the transoral-transclival operative approach for ventral extradural brain-stem or rostral cord compression include wound dehiscence (5.7%), pneumonitis (3.8%) and pulmonary embolus (1.9%) [11]. The probability of plate loosening complication in patient with irreducible atlantoaxial dislocation underwent TARP internal fixation was 6.5% [17]. The complications of transoral approach to the cervical spine include pulmonary complication (4.8%), wound complication (2.4%), death (2.4%) and venous thromboembolism (1.6%) [21]. The major complications rates of patients receiving surgery for chordomas were cerebrospinal fluid leakage (6.2%), velopharyngeal incompetence (3.1%), wound infection (3.1%), sepsis (3.1%), dysphagia (3.1%), failure of fixation (2.1%) and vertebral artery stroke (1%). The overall complication rates in patients with tumors were higher than in those without tumors, and this was probably due to a greater range of exposure and potential invasiveness of tumors [8]. Oropharyngeal complications (persistent pharynx discomfort, velopharyngeal insufficiency, pharynx ulcer, wound dehiscence, and infection), spinal canal complications (cerebrospinal fluid leak, meningitis secondary to infection, and epidural compressive hematoma), occipitocervical instability, pseudarthrosis, temporary quadriplegia, respiratory complications, and even death are the most common complications [1, 7, 11, 14, 15, 21, 22], which limit the use of transoral decompression.

Therefore, this study aimed to review the literature on the preoperative management of patients with upper cervical cord compression and postoperative complications associated with transoral decompression for upper cervical deformities, with a description on the risk factors, precautions, and treatments of these complications.

## Main text

### Preoperative management and postoperative complications

**Fig. 1:**
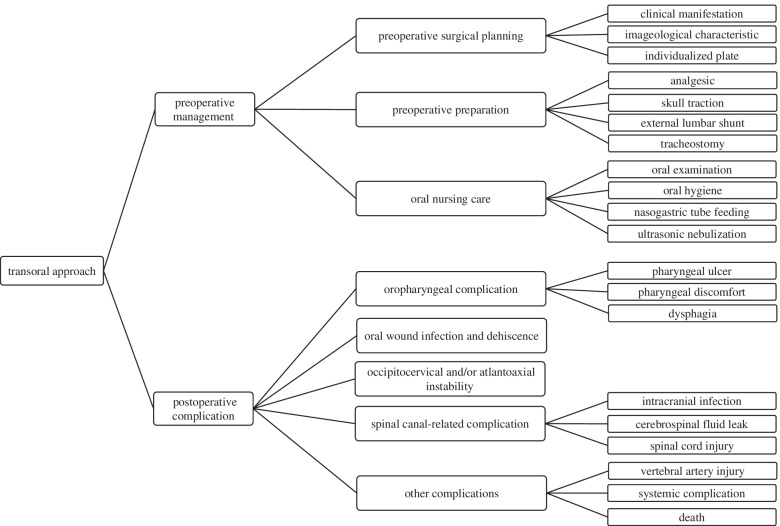
The preoperative management and the postoperative complications of transoral approach for the upper cervical deformities. Oral nursing care helps to reduce the occurrence of oropharyngeal complications, oral wound infection and dehiscence, as well as the occurrence of systemic complications such as pneumonia. Preoperative surgical planning and preoperative preparation play a role in reducing the occurrence of other complications

This study is focus on the preoperative management and the postoperative complications. Preoperative management include preoperative surgical planning, preoperative preparation, and oral nursing care. Postoperative complications include oropharyngeal complications, oral wound infection and dehiscence, occipitocervical and/or atlantoaxial instability, spinal canal-related complications, and other complications (Fig. 1). Oral nursing care helps to reduce the occurrence of oropharyngeal complications, oral wound infection and dehiscence, as well as the occurrence of systemic complications such as pneumonia. Preoperative surgical planning and preoperative preparation play a role in reducing the occurrence of other complications.

### Preoperative management

#### Preoperative surgical planning

The surgical approach for upper cervical deformities is dependent on the clinical manifestations and imageological characteristics. The American Spinal Injury Association (ASIA) neurological classification or the Japanese Orthopaedic Association (JOA) standard was used to evaluate preoperative neurological dysfunction, and visual analog scale (VAS) and Oswestry disability index (ODI) scores were used to evaluate pain. Flexion-extension radiographs could elucidate the stability of the atlantoaxial joint. In addition, CT scans could demonstrate the structure and degree of vertebral destruction. MRI could be used to reveal the extent of spinal cord compression and the size and location of tumors or infections. In a previous study, digital models, which were reconstructed according to CT scans, could be used to design individualized plates, and stereolithographic models could be used for the surgical simulation and custom-made implant fixation preoperatively [14].

#### Preoperative preparation

Most patients with upper cervical deformities suffer neck pain, neck movement restriction, and signs of myelopathy. The use of analgesics should be based on the VAS score and patients’ consent. For patients with atlantoaxial dislocation, a preoperative skull traction of 4–10 kg for 1–2 weeks is recommended, and the weight should be progressively increased from the minimal value of 4 kg to the maximum of 10 kg. Further, crown halo traction could be instituted for children who required skull traction [23]. In patients with irreducible atlantoaxial dislocation, continuous skull traction may not achieve satisfactory results [6, 24]. Approximately 64% (16/25) of patients with os odontoideum have been reported to have reduced subluxation after a preoperative skull traction [7]. Yang et al. [20] demonstrated that all patients underwent preoperative skull traction before the transoral revision surgeries. Moreover, preoperative skull traction may improve the clinical symptoms of spinal cord compression.

A preoperative external lumbar shunt enabled a surgeon to decrease the pressure of the dura mater and confirm and treat dura mater tear intraoperatively. However, preoperative placement of an external lumbar shunt may increase the risk of postoperative complications [1]. Therefore, the practice of external lumbar shunt placement should be considered by taking into account the individual situation and degree of spinal cord compression. Tracheostomy, another invasive procedure, could provide an appropriate and safe condition for transoral decompression, but it increased the incidence rate of infectious complications [25]. For patients with vagal, hypoglossal, or glossopharyngeal nerve dysfunction, tracheostomy should be considered despite the possibility of infection-related complications [26].

#### Oral nursing care

Preoperative oral preparation includes oral examination to exclude oropharyngeal infection and odontopathy, daily oral hygiene, and the use of Dobell’s solution or chlorhexidine collutory [27], 4–6 times daily for 3–5 days. Odontopathy, such as dental caries and gingivitis, should be treated preoperatively. Oral hygiene should be maintained preoperatively to prevent the occurrence of wound infection [28]. Nasogastric tube feeding is performed for 5–10 days postoperatively, and semiliquid feeding is arranged for the following week. Although the discomfort of nasogastric tube feeding is sometimes challenging, it is beneficial for the healing of the laryngopharyngeal wound and reduces the risk of infection [29]. Ultrasonic nebulization should be performed postoperatively to relieve the oropharyngeal discomfort and edema [30].

##### Surgical technique of transoral decompression for the upper cervical spine

**Fig. 2:**
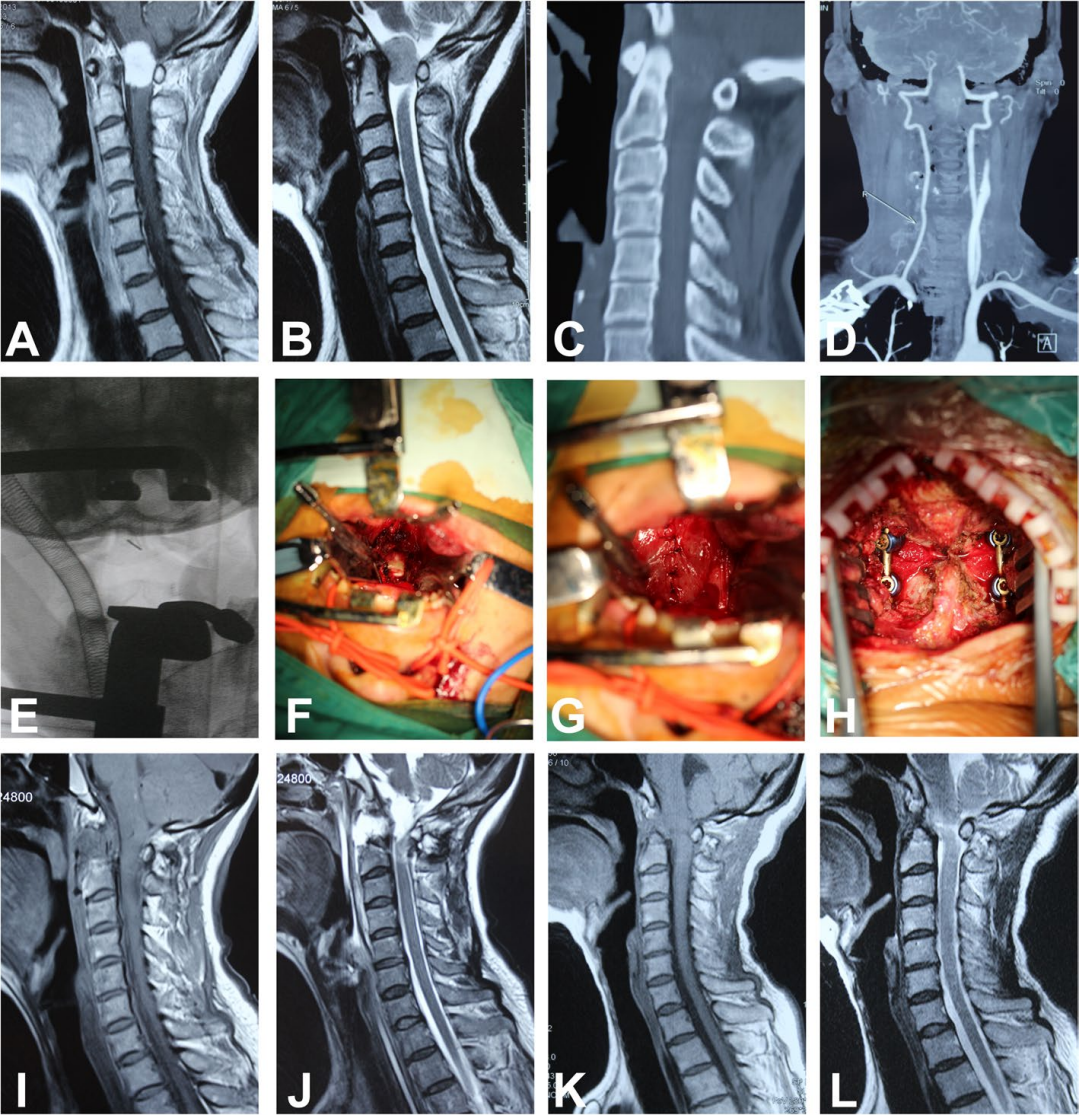
A 55-year-old woman presented with numbness and weakness of limbs and was diagnosis with basilar invagination. **A** and **B**: Preoperative sagittal T1-weighted and T2-weighted MR images show intraspinal tumor in the upper cervical spine. **C** and **D**: Preoperative sagittal CT scan and CT angiography of the upper cervical spine. **E**: Intraoperative fluoroscopy with locating pin. **F**: Intraoperative tumor exposure after spinal durotomy. **G**: Duraplasty after tumor excision. **H**: Posterior fusion and stabilization. **I** and **J**: Postoperative sagittal T1-weighted and T2-weighted MR images show that intraspinal tumor was resected and the spinal cord was sufficient decompressed. **K** and **L**: Sagittal T1-weighted and T2-weighted MR images at a follow-up of 3 months postoperatively and the patient could walk well after the operation

Essential preoperative preparation is completed. The transoral approach is performed with the assistance of adjustable retractor support, which could expand the mouth, depress the tongue and expose the posterior wall of the pharynx. An incision on the posterior pharyngeal wall is made. Then, the mucous membrane, pharyngeal constrictor, pharynx buccal fascia, vertebral muscles, and anterior longitudinal ligament are cut and retracted. The vertebral body of C2 is removed if necessary. The spinal tumor or abnormal vertebra is removed to achieve thorough decompression of spinal cord. Then suture or duraplasty is performed after transoral decompression. Posterior fusion are performed to reestablish the spinal stability. Transoral decompression and posterior fusion were performed for patients who presented with numbness and weakness of limbs and was diagnosis with basilar invagination (Fig. 2). Spinal meningioma was confirmed by postoperative pathology. Spinal cord decompression was achieved after surgery. Spinal cord function was recovering at 3-month follow-up, and the patient could walk well after the operation at subsequent follow-up.

#### Postoperative complications

##### Oropharyngeal complications

Oropharyngeal complications include pharyngeal ulcer, persistent pharyngeal discomfort, and dysphagia. Unsuitable implants and screw-loosening could cause pharyngeal discomfort, and even dysphagia [15, 17, 31]. Unsuitable implants could be regarded as unmatched size and location of implants. The space between the unsuitable plate and the surface of the atlas could eventually cause screw-loosening. Moreover, osteoporosis could also increase the risk of screw-loosening. Revision surgery should be performed for these patients to prevent worsening of symptoms. In addition, based on preoperative evaluation of the pathology and anatomical structure of lesions, extended transoral approaches might be selected. However, in a previous report, increased exposure was related to the risk of oropharyngeal complications [32]. To reduce oropharyngeal complications, surgical decisions involving the use of compatible and effective implants and the length of exposure according to clinical, anatomical, and radiographic manifestations are required. Intraoperatively, reconstruction of the atlantoaxial surface and implants could increase the attachment of the plate. Individualized transoral plates with a more suitable and compatible shape may have been designed for much better plate attachment. A previous research demonstrated that splitting of the soft palate should be avoided to decrease the incidence rate of oropharyngeal complications [33]. For patients with osteoporosis, anti-osteoporosis treatment should be arranged pre- and postoperatively in order to reduce the risk of screw-loosening. Further, transoral approach should be not performed for elderly patients with severe osteoporosis [17].

##### Oral wound infection and dehiscence

A retrospective review demonstrated that the incidence rate of oral wound infection was 3.5% (6/172) and that local oral wound infection was associated with dura mater tear and exposure of the internal plate fixation [34]. Another retrospective cohort study showed that the infection rate was 2.4% (3/126) [21]. In addition, Shousha et al. [35] concluded that the infection rate after a transoral surgery for the upper cervical spine was 3.6% (5/136). However, other studies reported unexpected oral wound infection rate, such as 0.1% (1/733) and 0% (0/29) [17, 23]. Preventive measures against the occurrence of infections and the limited sample size may be the reasons for the low wound infection rate. When comparing oral wound infection rates between most previous studies, different values were obtained, which were due to the included population, the pathogenesis of upper cervical deformities, and combined operation methods [7, 21, 23, 34–36]. Oral wound infection mostly occurs in the first 4 months postoperatively and could be treated after a one-stage debridement and suturing, but a delayed infection could occur several years later [35]. It was reported that an operation time of more than 4 h and a length of hospital stay of more than 5 days were associated with the occurrence of postoperative complications [21]. Moreover, rheumatic diseases and postoperative oncological therapy increased the risk of postoperative oral wound infection [35].

Perioperative broad-spectrum antibiotics for the oral flora could be used to reduce the risk of wound infection [18, 37]. In addition, professional oral nursing care could decrease the possibility of oral wound infection and dehiscence. When necessary, preoperative swabs should be taken from the oropharynx to exclude multiple-resistant pathogens [35]. Moreover, as mentioned above, preoperative tracheostomy and postoperative nasogastric feeding could facilitate wound healing and reduce the incidence rate of wound-related complications. After reaching a definite diagnosis of infection, antibiotics should be administered and internal fixations may be removed. Debridement and resuturing, as an effective method, is required for patients with oral wound dehiscence. In addition, venous nutritional support could enhance oral wound recovery, especially for patients with metabolic diseases or malnutrition [38].

##### Spinal canal-related complications

Intraoperative dural injury could cause postoperative cerebrospinal fluid leak. Dura mater tear and cerebrospinal fluid leak could be considered as risk factors for meningitis due to the connection between the oropharyngeal environment and cerebrospinal fluid. Severe spinal cord compression and obvious adhesion between the lesions and the dura would increase the possibility of intraoperative dural injury. To prevent the occurrence of dural injury, the lesion should be carefully resected. Moreover, obvious dura mater tear should be repaired as completely as possible intraoperatively. Fibrin glue, fascia, and bovine pericardium could be employed in the repair of dural injury [26, 39]. Additionally, patients with intracranial infection could be treated with antibiotics, and lumbar drainage and removal of internal implants could be added, as necessary.

Spinal cord injury after a transoral decompression could be caused by direct damage during decompression and indirect damage due to neck movement while changing the position for posterior fixation. Intraoperative monitoring could effectively reduce spinal cord injury associated with surgical procedures. With the development of surgical and assistive techniques, the incidence rate of intraoperative spinal cord injury is decreasing.

##### Occipitocervical and/or atlantoaxial instability

The preoperative and postoperative dynamic radiographs could be used to estimate the stability and reducibility of the occipitocervical junction. The instability may have been caused by the screw-loosening, poor bone fusion, and the inappropriate fixation methods, and could also result in unrecoverable neurological symptoms. Zhang et al. [40] demonstrated that transoral atlantoaxial reduction plate fixation with a C1-C2 joint cage could achieve better stability than posterior C1-C2 fixation using a finite element analysis. Regarding the transoral atlantoaxial reduction plate loosening, posterior fixation and fusion were recommended [15, 41]. Patients with os odontoideum who underwent transoral decompression may have occipitocervical instability; therefore, occipitocervical fusion should be chosen, instead of atlantoaxial fusion. Patients with basilar invagination, who underwent transoral decompression, may have recurrent craniocervical cord compression [42]. Revision surgery for transoral release with posterior reduction and fixation was recommended as a feasible and safe choice for patients with irreducible atlantoaxial dislocation who underwent previous surgeries [43]. The recurrence of upper cervical tumors could lead to implant displacement and instability, and a revision surgery would be necessary [35]. To maintain the stability of the upper cervical spine, a cervical collar should be used for 2–3 months. Additionally, osteoporosis may increase the possibility of screw-loosening in these patients; a halo vest should be fixed and maintained for 3 months [22, 44].

#### Other complications

##### Vertebral artery injury

Vertebral artery injury mostly occurred with the transoral decompression approach and was associated with posterior reduction and fixation; the artery was injured during the placement of transpedicular screws [36]. Preoperative computed tomography angiography (CTA) could be employed to clarify the location and trend of the vertebral artery. A previous study demonstrated the anatomical structure of the vertebral artery and nerves and their relationships [45]. Using the transoral approach, the surgeons’ familiarity with the anatomical structures of the vertebral artery may be directly related to the incidence of vertebral artery injury. When vertebral artery injury occurs intraoperatively, direct compression and use of hemostatic agents or induction of hemostasis are recommended, and a timely angiography could be performed to evaluate the degree and location of the vertebral artery injury. However, prevention of vertebral artery injury is better than its treatment.

##### Systemic complications

Systemic complications, such as respiratory infection, urinary system infection, cardiovascular system diseases, and pressure sores were not directly correlated with transoral surgery [46, 47]. The treatment principles should be consistent with the standards of related diseases.

##### Death

Death is the most serious complication. A previous study reported that perioperative mortality reached 11.1% [48]. Currently, with the improvement of perioperative management and surgical approaches, only a few deaths are directly associated with surgery involving transoral decompression. The most common cause of death is respiratory obstruction due to respiratory diseases or operative procedures [8, 15, 48, 49]. If a wound infection or meningitis is not treated promptly and effectively, the risk of death would increase.

## Conclusions

The effectiveness and safety of transoral decompression have been improved by constant development of the operative techniques and the use of advanced auxiliary diagnostic and therapeutic methods, with the understanding of the anatomical structure of the craniocervical joint. Oral wound infection and dehiscence and occipitocervical and/or atlantoaxial instability, as main complications, still hinder the use of the transoral approach.

Transoral decompression has been associated with individualized anterior implants that could be an effective method to manage atlantoaxial instability caused by basilar impression, craniovertebral junction meningioma, and Chiari malformation [14, 50, 51]. A cadaveric study confirmed that individualized templates may guide transoral C2 screw placement and decrease the rates of neurovascular complications [52]. Individualized anterior implants could improve the attachment and appropriateness of the plate and decrease the complication rates. In addition, the addictive manufacturing could be used to print the designed anterior plate, which could be manufactured according to the reconstruction of the upper cervical spine. This technique may be considered but still requires further studies to achieve a widespread application.

The less-invasive endoscopic endonasal approach allows similar degree of decompression in the craniocervical joint [53, 54]. This surgical technique could be an alternative or complementary approach for the decompression of the upper cervical spine [54–56]. It could decrease the incidence rates of dysphagia and respiratory complications [54]. Shkarubo et al. [25] also reported that the transoral approach with endoscopic assistance could significantly improve intraoperative visualization. The complication rate of the endoscopic endonasal approach has been reported as 76%; however, most of the complications were transient, such as dysphagia, cerebrospinal fluid leaks, pneumonia, and urinary tract infections [57]. However, the longer learning treatment, the lack of knowledge on the anatomical structure of the craniocervical joint, and the limited operating range may restrict the use of the endoscopic endonasal approach. Therefore, we believe that the transoral approach with endoscopic assistance indeed decreases the complication rate, although the disadvantages of the endoscopic endonasal approach still exist. In addition, it also provides an effective and safe method for transoral decompression. In recent years, spinal surgery robots came into being with the development of mechanical industry and computer navigation technology. The robots have the advantages of high precision, repeatability and endurance. They are able to push through the limitations of human functioning and further improve surgical accuracy [58]. At the same time, there are also some complications (postoperative hemorrhage, dysphagia and nerve injury) worth noting in transoral robotic surgery (TORS) [59, 60].

The use of individualized anterior implants and the less-invasive endoscopic endonasal approach has improved the effectiveness of transoral decompression and reduced the associated complications. Furthermore, with the advancement of personalized customization and endoscopic technique, the endoscopic endonasal approach combined with individualized plate may significantly decrease the complication rate of transoral decompression.

## Data Availability

Not applicable.

## References

[1] Amelot A, Terrier LM, Lot G (2018). Craniovertebral junction Transoral approach: predictive factors of complications. World Neurosurg.

[2] Perrini P, Benedetto N, Di Lorenzo N (2014). Transoral approach to extradural non-neoplastic lesions of the craniovertebral junction. Acta Neurochir.

[3] Kotil K, Kalayci M, Bilge T (2007). Management of cervicomedullary compression in patients with congenital and acquired osseous-ligamentous pathologies. J Clin Neurosci.

[4] Sar C, Eralp L (2001). Transoral resection and reconstruction for primary osteogenic sarcoma of the second cervical vertebra. Spine (Phila Pa 1976).

[5] Shaha AR, Johnson R, Miller J, Milhorat T (1993). Transoral-transpharyngeal approach to the upper cervical vertebrae. Am J Surg.

[6] Salunke P, Behari S, Kirankumar MV, Sharma MS, Jaiswal AK, Jain VK (2006). Pediatric congenital atlantoaxial dislocation differences between the irreducible and reducible varieties. J Neurosurg.

[7] Wu X, Wood K, Gao Y, Li S, Wang J, Ge T, Zhao B, Shao Z, Yang S, Yang C (2018). Surgical strategies for the treatment of os odontoideum with atlantoaxial dislocation. J Neurosurg Spine.

[8] Choi D, Melcher R, Harms J, Crockard A (2010). Outcome of 132 operations in 97 patients with chordomas of the craniocervical junction and upper cervical spine. Neurosurgery.

[9] Kerschbaumer F, Kandziora F, Klein C, Mittlmeier T, Starker M (2000). Transoral decompression, anterior plate fixation, and posterior wire fusion for irreducible atlantoaxial kyphosis in rheumatoid arthritis. Spine (Phila Pa 1976).

[10] Goel A, Bhatjiwale M, Desai K (1998). Basilar invagination a study based on 190 surgically treated patients. J Neurosurg.

[11] Hadley MN, Spetzler RF, Sonntag VK (1989). The transoral approach to the superior cervical spine. A review of 53 cases of extradural cervicomedullary compression. J Neurosurg.

[12] Sawin PD, Menezes AH (1997). Basilar invagination in osteogenesis imperfecta and related osteochondrodysplasias: medical and surgical management. J Neurosurg.

[13] Kingdom TT, Nockels RP, Kaplan MJ (1995). Transoral-transpharyngeal approach to the craniocervical junction. Otolaryngol Head Neck Surg.

[14] Shkarubo AN, Kuleshov AA, Chernov IV, Vetrile MS, Lisyansky IN, Makarov SN, Ponomarenko GP, Spyrou M (2018). Transoral decompression and stabilization of the upper cervical segments of the spine using custom-made implants in various pathologic conditions of the Craniovertebral junction. World Neurosurg.

[15] Yin QS, Li XS, Bai ZH, Mai XH, Xia H, Wu ZH, Ma XY, Ai FZ, Wang JH, Zhang K (2016). An 11-year review of the TARP procedure in the treatment of atlantoaxial dislocation. Spine (Phila Pa 1976).

[16] Ai FZ, Yin QS, Xu DC, Xia H, Wu ZH, Mai XH (2011). Transoral atlantoaxial reduction plate internal fixation with transoral transpedicular or articular mass screw of c2 for the treatment of irreducible atlantoaxial dislocation: two case reports. Spine (Phila Pa 1976).

[17] Yin QS, Ai FZ, Zhang K, Mai XH, Xia H, Wu ZH (2010). Transoral atlantoaxial reduction plate internal fixation for the treatment of irreducible atlantoaxial dislocation: a 2- to 4-year follow-up. Orthop Surg.

[18] Ai F, Yin Q, Wang Z, Xia H, Chang Y, Wu Z, Liu J (2006). Applied anatomy of transoral atlantoaxial reduction plate internal fixation. Spine (Phila Pa 1976).

[19] Yin Q, Ai F, Zhang K, Chang Y, Xia H, Wu Z, Quan R, Mai X, Liu J (2005). Irreducible anterior atlantoaxial dislocation one-stage treatment with a transoral atlantoaxial reduction plate fixation and fusion. Report of 5 cases and review of the literature. Spine (Phila Pa 1976).

[20] Yang J, Ma X, Xia H, Wu Z, Ai F, Yin Q (2014). Transoral anterior revision surgeries for basilar invagination with irreducible atlantoaxial dislocation after posterior decompression: a retrospective study of 30 cases. Eur Spine J.

[21] Steinberger J, Skovrlj B, Lee NJ, Kothari P, Leven DM, Guzman JZ, Shin J, Shrivastava R, Caridi JM, Cho SK (2016). Surgical morbidity and mortality associated with Transoral approach to the cervical spine. Spine (Phila Pa 1976).

[22] Menezes AH, VanGilder JC (1988). Transoral-transpharyngeal approach to the anterior craniocervical junction. Ten-year experience with 72 patients. J Neurosurg.

[23] Menezes AH (2008). Surgical approaches: postoperative care and complications “transoral-transpalatopharyngeal approach to the craniocervical junction”. Childs Nerv Syst.

[24] Hao DJ, He BR, Wu QN (2009). One-stage anterior release and reduction with posterior fusion for treatment of irreducible atlantoaxial dislocation. Orthop Surg.

[25] Shkarubo AN, Andreev DN, Konovalov NA, Zelenkov PV, Lubnin AJ, Chernov IV, Koval KV (2017). Surgical treatment of Skull Base tumors, extending to Craniovertebral junction. World Neurosurg.

[26] Hsu W, Wolinsky JP, Gokaslan ZL, Sciubba DM (2010). Transoral approaches to the cervical spine. Neurosurgery.

[27] Varoni E, Tarce M, Lodi G, Carrassi A (2012). Chlorhexidine (CHX) in dentistry: state of the art. Minerva Stomatol.

[28] Gondo T, Fujita K, Nagafuchi M, Obuchi T, Ikeda D, Yasumatsu R, Nakagawa T (2020). The effect of preventive oral care on postoperative infections after head and neck cancer surgery. Auris Nasus Larynx.

[29] Plonowska KA, Ochoa E, Zebolsky AL, Patel N, Hoppe KR, Ha PK, Heaton CM, Ryan WR (2021). Nasogastric tube feeding after transoral robotic surgery for oropharynx carcinoma. Am J Otolaryngol.

[30] Marseu K, Slinger P (2016). Peri-operative pulmonary dysfunction and protection. Anaesthesia.

[31] Wang C, Yan M, Zhou HT, Wang SL, Dang GT (2006). Open reduction of irreducible atlantoaxial dislocation by transoral anterior atlantoaxial release and posterior internal fixation. Spine (Phila Pa 1976).

[32] Youssef AS, Sloan AE (2010). Extended Transoral Approaches: Surgical Technique and Analysis. Neurosurgery.

[33] Jones DC, Hayter JP, Vaughan ED, Findlay GF (1998). Oropharyngeal morbidity following transoral approaches to the upper cervical spine. Int J Oral Maxillofac Surg.

[34] Yin Q, Xia H, Wu Z, Ma X, Ai F, Zhang K, Wang J, Zhang T, Bai Z, Wang Z (2016). Surgical site infections following the Transoral approach: a review of 172 consecutive cases. Clin Spine Surg.

[35] Shousha M, Mosafer A, Boehm H (2014). Infection rate after Transoral approach for the upper cervical spine. Spine.

[36] Xu ZW, Liu TJ, He BR, Guo H, Zheng YH, Hao DJ (2015). Transoral anterior release, odontoid partial resection, and reduction with posterior fusion for the treatment of irreducible atlantoaxial dislocation caused by odontoid fracture malunion. Eur Spine J.

[37] Mooney MA, Oppenlander ME, Kakarla UK, Theodore N (2017). Tumoral calcinosis of the craniovertebral junction as a cause of dysphagia with treatment by transoral decompression: case report. J Neurosurg Spine.

[38] Lee SH, Park K, Kong DS, Kim ES, Eoh W (2010). Long-term follow up of transoral anterior decompression and posterior fusion for irreducible bony compression of the craniovertebral junction. J Clin Neurosci.

[39] Perrini P, Benedetto N, Guidi E, Di Lorenzo N (2009). Transoral approach and its superior extensions to the craniovertebral junction malformations: surgical strategies and results. Neurosurgery.

[40] Zhang B, Liu H, Cai X, Wang Z, Xu F, Liu X, Wang H, Kang H, Ding R (2016). Biomechanical comparison of modified TARP technique versus modified Goel technique for the treatment of basilar invagination: a finite element analysis. Spine (Phila Pa 1976).

[41] Dickman CA, Locantro J, Fessler RG (1992). The influence of transoral odontoid resection on stability of the craniovertebral junction. J Neurosurg.

[42] Goel A (2005). Progressive basilar invagination after transoral odontoidectomy treatment by atlantoaxial facet distraction and craniovertebral realignment. Spine (Phila Pa 1976).

[43] Tan M, Jiang X, Yi P, Yang F, Tang X, Hao Q, Zhang G (2011). Revision surgery of irreducible atlantoaxial dislocation: a retrospective study of 16 cases. Eur Spine J.

[44] Xia H, Yin Q, Ai F, Ma X, Wang J, Wu Z, Zhang K, Liu J, Xu J (2014). Treatment of basilar invagination with atlantoaxial dislocation: atlantoaxial joint distraction and fixation with transoral atlantoaxial reduction plate (TARP) without odontoidectomy. Eur Spine J.

[45] Wang Z, Xia H, Wu Z, Ai F, Xu J, Yin Q (2014). Detailed anatomy for the transoral approach to the craniovertebral junction: an exposure and safety study. J Neurol Surg B Skull Base.

[46] Mouchaty H, Perrini P, Conti R, Di Lorenzo N (2009). Craniovertebral junction lesions: our experience with the transoral surgical approach. Eur Spine J.

[47] Kandziora F, Mittlmeier T, Kerschbaumer F (1999). Stage-related surgery for cervical spine instability in rheumatoid arthritis. Eur Spine J.

[48] Jain VK, Behari S, Banerji D, Bhargava V, Chhabra DK (1999). Transoral decompression for craniovertebral osseous anomalies: perioperative management dilemmas. Neurol India.

[49] Landeiro JA, Boechat S, Christoph DH, Gonçalves MB, Castro I, Lapenta MA, Ribeiro CH (2007). Transoral approach to the craniovertebral junction. Arq Neuropsiquiatr.

[50] Shkarubo AN, Kuleshov AA, Chernov IV, Vetrile MS (2017). Transoral decompression and anterior stabilization of atlantoaxial joint in patients with basilar impression and Chiari malformation type I: a technical report of 2 clinical cases. World Neurosurg.

[51] Li X, Ai F, Xia H, Wu Z, Ma X, Yin Q (2014). Radiographic and clinical assessment on the accuracy and complications of C1 anterior lateral mass and C2 anterior pedicle screw placement in the TARP-III procedure: a study of 106 patients. Eur Spine J.

[52] Li XS, Wu ZH, Xia H, Ma XY, Ai FZ, Zhang K, Wang JH, Mai XH, Yin QS (2014). The development and evaluation of individualized templates to assist transoral C2 articular mass or transpedicular screw placement in TARP-IV procedures: adult cadaver specimen study. Clinics.

[53] Chibbaro S, Ganau M, Cebula H, Nannavecchia B, Todeschi J, Romano A, Debry C, Proust F, Olivi A, Gaillard S (2019). The Endonasal endoscopic approach to pathologies of the anterior Craniocervical junction: analytical review of cases treated at four European neurosurgical Centres. Acta Neurochir Suppl.

[54] Morales-Valero SF, Serchi E, Zoli M, Mazzatenta D, Van Gompel JJ (2015). Endoscopic endonasal approach for craniovertebral junction pathology: a review of the literature. Neurosurg Focus.

[55] Hussain I, Schwartz TH, Greenfield JP (2018). Endoscopic Endonasal approach to the upper cervical spine for decompression of the Cervicomedullary junction following Occipitocervical fusion. Clin Spine Surg.

[56] Visocchi M, Di Martino A, Maugeri R, Gonzalez Valcarcel I, Grasso V, Paludetti G (2015). Videoassisted anterior surgical approaches to the craniocervical junction: rationale and clinical results. Eur Spine J.

[57] Zwagerman NT, Tormenti MJ, Tempel ZJ, Wang EW, Snyderman CH, Fernandez-Miranda JC, Gardner PA (2018). Endoscopic endonasal resection of the odontoid process: clinical outcomes in 34 adults. J Neurosurg.

[58] Zamorano L, Li Q, Jain S, Kaur G (2004). Robotics in neurosurgery: state of the art and future technological challenges. Int J Med Robot.

[59] Sethi RKV, Chen MM, Malloy KM (2020). Complications of Transoral robotic surgery. Otolaryngol Clin N Am.

[60] Yee S (2017). Transoral robotic surgery. AORN J.

